# Mathematical anxiety is associated with general rather than specific weakness in attention

**DOI:** 10.3389/fpsyg.2026.1763006

**Published:** 2026-05-13

**Authors:** Sarit Ashkenazi, Yehudit Danan

**Affiliations:** The Seymour Fox School of Education, The Hebrew University of Jerusalem, Jerusalem, Israel

**Keywords:** attention control, attentional mechanisms, flanker task, mathematical anxiety, math-related stimuli, visuospatial working memory

## Abstract

**Introduction:**

Math anxiety (MA) is a feeling of tension and anxiety that interferes with an individual’s ability to solve mathematical problems. While some theories suggest that MA is based on a general weakness in spatial abilities, others propose that attention is only disrupted due to exposure to negative stimuli.

**Methods:**

To resolve this conflict, we employed a spatial flanker task with negative, neutral, or mathematical pictures as irrelevant distractors. We examined both verbal MA (using a self-report questionnaire) and nonverbal MA (using a rating of math-related pictures).

**Results:**

Verbal MA was associated with a larger location congruity effect. Conversely, nonverbal MA modulated the response to negative distractors: high nonverbal MA participants were less affected by negative pictures than low nonverbal MA participants. While low nonverbal MA participants showed a disengagement bias toward the negative picture, high MA participants showed the regular location congruity effect regardless of emotional valence.

**Discussion:**

The result from verbal MA assessments supports the presence of spatial/attentional deficits associated with MA.

## Introduction

Math anxiety (MA) is defined as a feeling of tension and anxiety that interferes with an individual’s ability to manipulate numbers and solve mathematical problems in a wide variety of everyday and academic situations (Richardson and Suinn, 1972). MA is related to avoidance of math-related topics (Maloney and Beilock, 2012), as well as to decreased motivation to engage in math.

A few theoretical explanations have been proposed for the etiology and preservation of MA, which vary in relation to the specific effect of mathematical stimuli on performance. While some of the theories suggest that MA, though similar to other anxiety disorders, is based on deficits that are non-specific to mathematical contexts, such as attentional, working memory, or spatial deficits (Danan and Ashkenazi, 2022; González-Gómez et al., 2023), other theories suggest that attention or working memory is only disrupted in high MA (HMA) participants because of exposure to math stimuli (Rubinsten et al., 2015; Suárez-Pellicioni et al., 2015).

The first line of studies suggests that MA originates from a specific cognitive weakness. For example, the spatial theory of MA suggests that the preverbal representation of quantities is spatial in nature, and children with low spatial abilities have weaknesses in the development of these preverbal representations, resulting in anxiety later on (Ashkenazi and Danan, 2017; Georges et al., 2016). Moreover, converging evidence links mathematical and spatial abilities (Lauer and Lourenco, 2016). In line with this theory, multiple studies indicated that HMA participants are characterized by spatial working memory deficits (e.g., Ashkenazi et al., 2024).

In contrast to the spatial theory, other theories have suggested that individuals with MA are characterized by reduced cognitive abilities such as executive functions, that are independent of the numerical task performed (Hopko et al., 1998; Mammarella et al., 2015). For example, researchers have also identified inhibition deficits in individuals with HMA (Hopko et al., 1998; Mammarella et al., 2015). Specifically, Colomé et al. (2023) used a Stroop task among participants with HMA or low MA (LMA) to assess both reactive and proactive control. Proactive and reactive control are distinguished by the proportion of congruent to incongruent trials within a given block. Specifically, proactive control is elicited in blocks containing a high ratio of incongruent trials, whereas reactive control is utilized in blocks with a low ratio of incongruent trials. The results showed that among participants with HMA, the congruency effect increased as MA increased (Colomé et al., 2023). Good inhibitory ability makes it possible to clear working memory from task-irrelevant distractors (Kattner, 2021). Hence, deficits in inhibition may be the origin of weakened working memory abilities in participants with HMA.

The second line of theories suggests that the attention or executive functions of participants with HMA are disrupted only when exposed to math-related stimuli. Attentional selectivity toward math stimuli has been proposed as a mechanism that may contribute to initiating, maintaining, or aggravating MA (Rubinsten et al., 2015; Suárez-Pellicioni et al., 2015). Clinical and subclinical populations with anxiety tend to bias their attention automatically toward or away from threats (Okon-Singer, 2018).

With relevance to MA, in a modified dot-probe task, participants were asked to decide whether one or two dots were shown on the screen. Prior to seeing the dots, participants were shown a prime (math-related/unrelated word). Individuals with MA reacted faster when the probe was in the location of the math-related stimuli, suggesting the existence of an attentional bias toward math-related stimuli among participants with HMA (Rubinsten et al., 2015). In another study, Pizzie and Kraemer (2017) found attentional avoidance of math-related stimuli. In this study, the dot-probe task was administered to participants with HMA, using three possible cues: negative pictures (such as injuries), neutral valence pictures (such as houses), and mathematical equations. Participants in the HMA group exhibited attention allocation toward negative pictures, whereas they showed attentional avoidance of the mathematical equations (Pizzie and Kraemer, 2017). Attentional bias in individuals with HMA was also found in the emotional Stroop task. In this task, participants were asked to press a key to indicate the color of the word’s ink, while ignoring the word’s meaning. When the word was math-related, participants with HMA were slower to respond and had higher error rates than those with LMA (Suárez-Pellicioni et al., 2015).

## The current study

To resolve the conflict between the two lines of theories, a recent study examined whether MA is associated with deficits in selective attention expressed in attentional bias or with reduced attentional control or executive function. In this spatial probe task, an initial figure appeared on either the left or right side of the screen, followed by either a math equation or a neutral stimulus. A second figure then appeared on either side of the screen, and participants were required to indicate whether the two figures were identical or different. By utilizing math equations as task-irrelevant stimuli, the results revealed no significant differences between groups regarding attentional bias, specifically, the selective attention toward math equations compared to neutral stimuli. Consequently, this study provides evidence against an attentional bias for mathematical symbols during visuospatial orienting among participants with HMA. Rather, those in the HMA group were generally slower in attentional tasks (González-Gómez et al., 2023). In a recent study, we used a spatial flanker task in MA participants, and similar to González-Gómez et al. (2023), we discovered that the HMA group showed a larger spatial congruity effect (difference in reaction time between trials that the spatial location of the target and distractors were not identical compared to trials where the spatial location of the target and distractors were identical) compared to LMA participants, which was then found to be associated with reduced visuospatial working memory abilities (Ashkenazi et al., 2024).

The current study modified the paradigm proposed by Lichtenstein-Vidne and colleagues (Ashkenazi et al., 2024; Lichtenstein-Vidne et al., 2012; Lichtenstein-Vidne et al., 2017) to examine how MA affects orienting of attention in a spatial flanker task to irrelevant math-related, negative, or neutral pictures. Lichtenstein-Vidne et al. (2017) demonstrated that individuals with generalized anxiety frequently exhibit an attentional bias toward task-irrelevant, negatively valenced distractors. To investigate the attentional mechanisms underlying HMA, we adapted this paradigm by incorporating math-related images as task-irrelevant distractors. In this paradigm, participants were instructed to indicate whether a target picture appeared in the upper or lower part of the screen. Two peripheral distracting pictures also appeared, either in the location next to the target picture (congruent) or in the opposite location (incongruent)—a procedure that examines spatial processing. Crucially, the emotional context of the distractor was manipulated between negative, math-related, or neutral categories. Participants with anxiety disorders often exhibit attentional bias toward negative, irrelevant pictures. If MA is similar to other anxiety disorders, then participants with HMA are expected to show an attentional bias toward negative and mathematical pictures (Lichtenstein-Vidne et al., 2012; Lichtenstein-Vidne et al., 2017). However, if MA is based on a visuospatial working memory weakness, one can expect a larger congruity effect among HMA individuals, regardless of distractor valence, compared to low MA individuals (Ashkenazi et al., 2024).

Hence, the current study had several goals. First, we aimed to explore the specific manifestation of attentional and spatial mechanisms in MA using the modified spatial flanker task. Second, to examine the influence of negative valence pictures alongside mathematically related pictures, given that prior work found differences in attentional mechanisms toward negative or mathematical stimuli in HMA participants (Pizzie and Kraemer, 2017). Finally, most other studies regarding MA used only verbal self-reports. In the current study, we measured nonverbal MA using ratings of our newly created set of math-related pictures. Since MA is a specific anxiety of mathematical contexts and has been found to be correlated with general anxiety, it was necessary to control for individual differences in emotional ratings. Therefore, the nonverbal MA score was calculated as the mean negative rating of math-related pictures minus the mean rating of neutral pictures. Accordingly, the average rating of the neutral pictures was defined as the emotional baseline. We then tested the correlations between attentional bias, this new measure, and multiple scores from verbal measures of MA. This nonverbal MA measure provides a novel approach compared to earlier studies, which relied primarily on explicit verbal measures. A fundamental limitation of verbal self-report measures, such as the Mathematics Anxiety Rating Scale (MARS), and the Mathematics Anxiety Questionnaire (MAQ), is their reliance on subjective appraisal. This reliance introduces vulnerabilities to introspective bias, social desirability, and the confounding influence of actual mathematical competence. In contrast, our newly developed nonverbal assessment is significantly less susceptible to these biases. Ashkenazi et al. (2024) validated a set of math-related pictures, finding that participants with HMA rated these images as significantly more anxiety-provoking than did those with low MA. Similarly, Linares et al. (2025) demonstrated that math-related imagery can effectively elicit anxiety in individuals with HMA. Collectively, this literature demonstrates that math-related imagery serves as a valid and effective tool for assessing nonverbal MA.

## Hypotheses

We hypothesize that we will find a larger congruity effect in the spatial flanker task (the difference between reaction times for congruent and incongruent trials) as the verbal MA increases (Ashkenazi et al., 2024). Because negative pictures automatically capture attention, we expect the congruity effect with negative distractors to be smaller, or even reversed, compared to the congruity effect with neutral distractors. The nonverbal measurement of MA in this study will provide additional value compared to the traditional self-report measurements of MA currently employed. We predict a weak but meaningful correlation between the nonverbal and self-report measurements of MA.

## Methods

### Sample and procedure

Forty-eight participants took part in the study (15 males and 27 females; mean age 23.26 years, SD 2.37 after exclusion). All individuals were students at the Seymour Fox School of Education at the Hebrew University, and participated to receive course credit. Of these, six were excluded due to low accuracy in the task (lower than 80% in one of the conditions). All participants provided informed consent, then completed the main Math-Related Modified Flanker Task (MRMFT), which included negative, neutral, and mathematical pictures as non-relevant distractors. This was followed by three questionnaires. Last, the participants rated the mathematical and neutral pictures. The experiment was conducted online. The participants were first-year Education students enrolled in an Introduction to Psychology course. They participated in the experiment to receive course credit. Initially, they signed up using Sona Systems,^1^ an online experiment management software. Upon registering, participants received a link to the Gorilla platform via the system.

To validate our innovative approach to assessing nonverbal MA, we included established measures for comparison. Accordingly, we administered a widely used verbal MA questionnaire (MARS) alongside the MAQ, which examines both the cognitive and emotional aspects of verbal MA. We aimed to determine whether our nonverbal measure correlated with these verbal measures. Furthermore, the STAI-S was administered to explicitly rule out the possibility that nonverbal MA is merely reflecting general anxiety.

### Tools for assessing MA

#### Mathematics anxiety rating scale—short version

We used a short version of the Mathematics Anxiety Rating Scale (MARS) that examines stable mathematical anxiety (Suinn and Winston, 2003). The short version, which comprises 30 items selected from the full MARS (Alexander and Martray, 1989), has been shown to provide reliable and valid information. A Cronbach alpha of 0.96 indicated high internal consistency, with test–retest reliability for the MARS 30-item of 0.90 (*p* < 0.001) (Alexander and Martray, 1989). The MARS assesses an individual’s level of math apprehension and anxiety on a 1–5 Likert scale, with participants indicating how anxious they would be made by various settings and experiences (e.g., “Taking the math section of a standardized test” or “walking to math class.”). A higher rating on the scale corresponds to a greater degree of math anxiety in daily life.

#### Math anxiety questionnaire

The Math Anxiety Questionnaire (MAQ) was used for specific assessment of the emotional and cognitive components of MA (Kazelskis et al., 2000; Wigfield and Meece, 1988). The MAQ examines stable mathematical anxiety, including its cognitive and affective factors, with the total score representing the combination of the two scores (Thomas and Dowker, 2000). In a standardization study, the internal consistency (Cronbach’s alpha) was reported to vary between 0.83 and 0.91 for the entire questionnaire, depending on the age group (Krinzinger et al., 2007). The MAQ includes 11 questions, each answered on a 7-point scale. The items focus on negative affective reactions to performing math activities in school (e.g., “Taking math tests scares me”) and on students’ concerns about their mathematics performance (e.g., “In general, how much do you worry about how well you are doing in math?”). A higher rating on the scale corresponds to a greater degree of math anxiety in daily life.

#### Nonverbal measurement of MA

Every participant rated the mathematical pictures or a neutral picture according to the level of anxiety that each of the pictures evoke in the individual. During the rating task, participants were exposed to pictures in random order for unlimited time. Then, three questions appeared, one after the other, for an unlimited time on a Likert scale between 1 (not at all) to 9 (very much). (1) how much the picture evokes a sense of inconvenience, arousal, or preparedness, (2) how much the picture evokes a sense of lack of control, and (3) how much the picture evokes a sense of anxiety. We calculated the mean score for each set of questions (math-related or neutral) across the three questions. To control for individual differences in emotional ratings, each individual’s average rating of the neutral pictures served as the baseline. Accordingly, the average rating of the mathematical pictures minus the average rating of the neutral pictures was defined as the measure of nonverbal MA. There were 48 mathematically related pictures. The majority of these pictures showed a teacher giving a math lecture, with or without students solving problems at the board. Others depicted a math test, a mathematical equation on a blackboard, or a student actively solving a problem on the class board. There were also 51 neutral pictures showing students in day-to-day activities or trips and empty classes. The instructions for the rating phase were “In the study, we are interested in how people feel toward different events, presented in pictures. The pictures, which will be projected before you, show various events occurring in a classroom.

You are asked to review the different pictures appearing on the screen and to rate each picture in terms of—“how it made you feel, when you looked at the picture.” In this study, there is no right or wrong answer—we ask you to respond to the picture as honestly and directly as you can. Before you, three scales will appear one after the other. On each scale, a continuum of the level of feeling is presented between 1 and 9. You will need to rate every picture you view according to the three scales. Scale A—Examines feelings between high alertness and arousal (high numbers) to calmness and relaxation (low numbers). Scale B—Tests the feeling of control (high numbers) in the face of the situation versus a lack of control (low numbers). Scale C—Tests the feeling of anxiety that the picture evokes in you (high numbers) versus a lack of anxiety (low numbers). These instructions were given in the task. In each trial a fixation point was presented for 250 ms, followed by the picture, which was presented for an unlimited time until the participants pressed the continue button. Then Scale A was presented with the question regarding inconvenience with a scale between 1 and 9, then Scale B was presented with the question about the lack of control with a scale between 1 and 9, and then Scale C was presented with the question about the level of anxiety with a scale between 1 and 9. Supplementary Table S1 presents the mean ratings for each participant.

#### The state scale of the state–trait anxiety inventory

The STAI (Kendall et al., 1976) is one of the most frequently used measures in applied psychology research to test participants’ current feelings, and is considered to be a reliable and sensitive measure of anxiety. Participants answer each item on a 4-point scale, ranging from “not at all” to “very much.” The items ask about the participants’ current feelings, including, for example, stress or calmness. The six-item short-form of the STAI yields scores similar to those obtained using the full 20-item STAI (*α* = 0.82) (Marteau and Bekker, 1992). The short form has acceptable reliability. We have calculated the average score on the STAI negative questions (e.g., level of stress) and calculated the average score on the STAI positive questions (e.g., level of calmness). The STAI negative questions were significantly correlated with the MARS *r*(46) = 0.36, *p* = 0.019, negatively correlated with the STAI positive, *r*(46) = −0.55, *p* = 0.001. The STAI negative was not significantly related to any of the other nonverbal or verbal measurements of MA. The STAI positive was not significantly correlated with any of the MA measurements. We also created a composite score of the STAI by averaging the negative STAI and the opposite of the positive STAI (the answer 1 was transformed to 4, 2 was transformed to 3, 3 was transformed to 2, and 4 was transformed to 1). This score did not correlate significantly with any of the measurements (maximum *r* = 0.26, *p* = 0.1 with MAQ emotional). The score of STAI did not correlate with any of the reaction times in the different conditions and the congruity effect in neutral, math, or negative valence (minimum *p* = 0.21, *r* = 0.2).

#### Math-related modified flanker task

**Figure 1:**
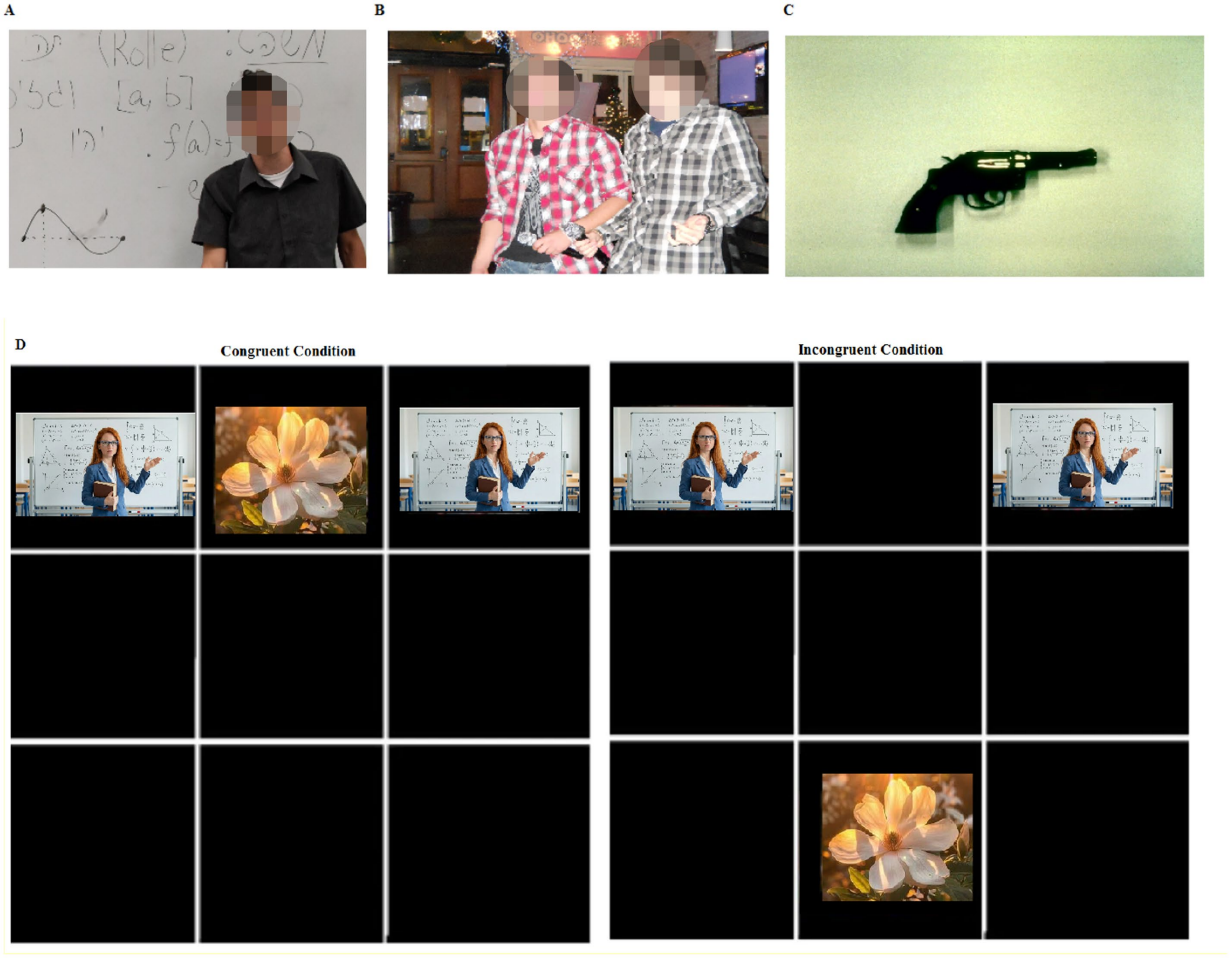
Examples of math-related pictures **(A)**, neutral pictures **(B)**, and negative pictures **(C)** used in the experiment. **(D)** Sample presentation in the flanker task for a location-congruent condition (left side) and a location-incongruent condition (right side). In location-congruent trials, the central target picture and the distracting peripheral pictures were all presented in the same row (either upper or lower). In location-incongruent trials, the central picture was presented in the opposite row (i.e., upper or lower) relative to the distracting peripheral pictures (i.e., lower or upper, respectively). In all conditions, participants were instructed to ignore the peripheral pictures and respond according to the location of the central picture.

The MRMFT task tests two components: (1) task-relevant spatial location, and (2) task-irrelevant math-related or negative content of distractors (Cronbach’s Alpha = 0.98). The paradigm was based on several studies (Lichtenstein-Vidne et al., 2007; Lichtenstein-Vidne et al., 2012; Lichtenstein-Vidne et al., 2017). In the MRMFT, we add to the negative emotional pictures a set of math-related pictures created in our lab. Participants were shown a black screen divided into 9 squares (3 rows × 3 columns). Each trial started with a fixation cross-hair presented in the central square for 500 ms, followed by three pictures, which appeared at the same time: a single neutral target picture and two distracting pictures. The target picture appeared in the middle square in the top or bottom row. The distracting pictures were two identical pictures that appeared on the peripheral columns of the upper or lower rows. Therefore, the location of the peripheral distractors could be congruent or incongruent with the location of the central target picture. Participants were asked to indicate, via keypress, whether the target appeared in the upper or lower part of the screen (see Figure 1).

The target picture was taken from the International Affective Picture System (IAPS)^2^ (Lane et al., 1997) and alternated between two examples of flowers (see Figure 1 for an example). The math-related distractors were pictures taken from the set of 48 math-related pictures as described above, such as a picture of a lecturer teaching math in a university course, a university student being asked to solve a mathematical equation on the board, and a student engaging in a mathematical task. Pictures were selected based on ratings done in a pilot study (Ashkenazi et al., 2024). The neutral distractors included situations from students’ lives, such as two students walking on campus. The negative pictures were taken from the IAPS (Lane et al., 1997). RT and accuracy were measured using Gorilla software from the target onset until the reaction. Cronbach’s alpha for the modified math-related task was very high = 0.967 (Ashkenazi et al., 2024).

### Analysis

Prior to the analysis, responses shorter than 150 ms or greater than 3,000 ms were excluded. Then a four-way repeated-measures mixed ANCOVA on the RTs was conducted for the correct trials, with location congruency (congruent and incongruent) and distractor valence (neutral negative or math-related) as within-subjects factors, and MARS rating and nonverbal MA as continuous variables.

These were all the possible interactions in the current analysis: (1) congruency and verbal MA, (2) congruency and non-verbal MA, (3) congruency and valence, (4) valence and verbal MA, (5) valence and non-verbal MA, (6) verbal MA and nonverbal MA, (7) congruency, valence, and verbal MA, (8) congruency, valence, and nonverbal MA (9) congruency, nonverbal MA, and verbal MA, (10) valence, verbal MA and nonverbal MA, (11) congruity, valence, verbal MA, and nonverbal MA.

Where applicable, we will report the Vovk-Sellke Maximum p-Ratio (VS-MPR). This metric provides a cautious, Bayesian interpretation of *p*-values, assisting researchers in avoiding the overstatement of marginally significant results. Following standard interpretation guidelines, VS-MPR values were classified as indicating anecdotal (1–3), substantial (3–10), strong (10–30), very strong (30–100), or decisive (>100) evidence for the alternative hypothesis.

## Results

### Correlations between MA tests

Because the MA tests are expected to look at a similar theoretical structure, we examined the correlations between and within these tests. First, a high correlation emerged between the emotional and cognitive parts of the MAQ, *r*(46) = 0.77, *p* = 0.01. Second, a high correlation emerged between the MARS and MAQ cognitive scale *r*(46) = 0.67, *p* = 0.01, and between the MARS and MAQ emotion scale *r*(46) = 0.58, *p* = 0.01, as well as between MARS and MAQ general scale *r*(46) = 0.66, *p* = 0.01. These high correlations add to the validity of the two questionnaires. Nevertheless, the correlations are not at a ceiling level, suggesting they do examine different aspects of MA.

Importantly, all the questionnaires or self-report measurements were significantly correlated with the score in the nonverbal measurement of MA. Specifically, the nonverbal MA score was positively related to the MARS *r*(46) = 0.31, *p* = 0.03, positively related to the MAQ emotion, *r*(46) = 0.48, *p* = 0.001, positively related to the MAQ cognitive, *r*(46) = 0.37, *p* = 0.009 and positively related to the MAQ general, *r*(46) = 0.46, *p* = 0.001. This indicates that nonverbal MA is significantly associated with verbal MA, mostly with MAQ emotions.

### Descriptive statistics

Table 1 shows the descriptive statistic of all the variables and includes correlations between each of the variables and verbal MA (MARS) or nonverbal MA (math-related negativity), whereas Table 2 shows the correlations between all the variables in the experiment.

**Table 1:** Descriptive statistics for all the tasks and questionnaires and correlation with verbal MA (MARS) or nonverbal MA (math-related negativity).

Task	Minimum	Maximum	Mean	SD	r MARS	r Math- related negativity
MARS	1.07	4.75	2.80	0.88	1	0.33*
MAQ emotional	1.14	7.00	4.35	1.74	0.58**	0.49**
MAQ cognitive	1.00	6.75	4.30	1.59	0.69**	0.46**
MAQ general	1.32	6.66	4.33	1.57	0.67**	0.46**
Math-related negativity	−0.47	6.37	2.04	−0.47	0.33*	1
STAI-negative	1.00	3.67	2.01	0.77	0.36*	−0.09
STAI-positive	1.00	4.00	2.36	0.79	−0.01	−0.03

*p < 0.05, **p < 0.01.

**Table 2:** Correlations between all the variables.

Task	1	2	3	4	5	6	7
(1) MARS	1	0.58***	0.66***	0.66***	0.37*	−0.03	0.31*
(2) MAQ emotion		1	0.77***	0.95***	0.24	−0.22	0.48**
(3) MAQ cognitive			1	0.94***	0.27	−0.13	0.37**
(4) MAQ general				1	0.27	−0.19	0.46**
(5) STAI negative					1	−0.53**	−0.03
(6) STAI positive						1	−0.05
(7) Nonverbal MA							1

*p < 0.05, **p < 0.01, ***p < 0.001 (Ashkenazi et al. 2024).

### MRMFT: reaction time analysis

**Figure 2:**
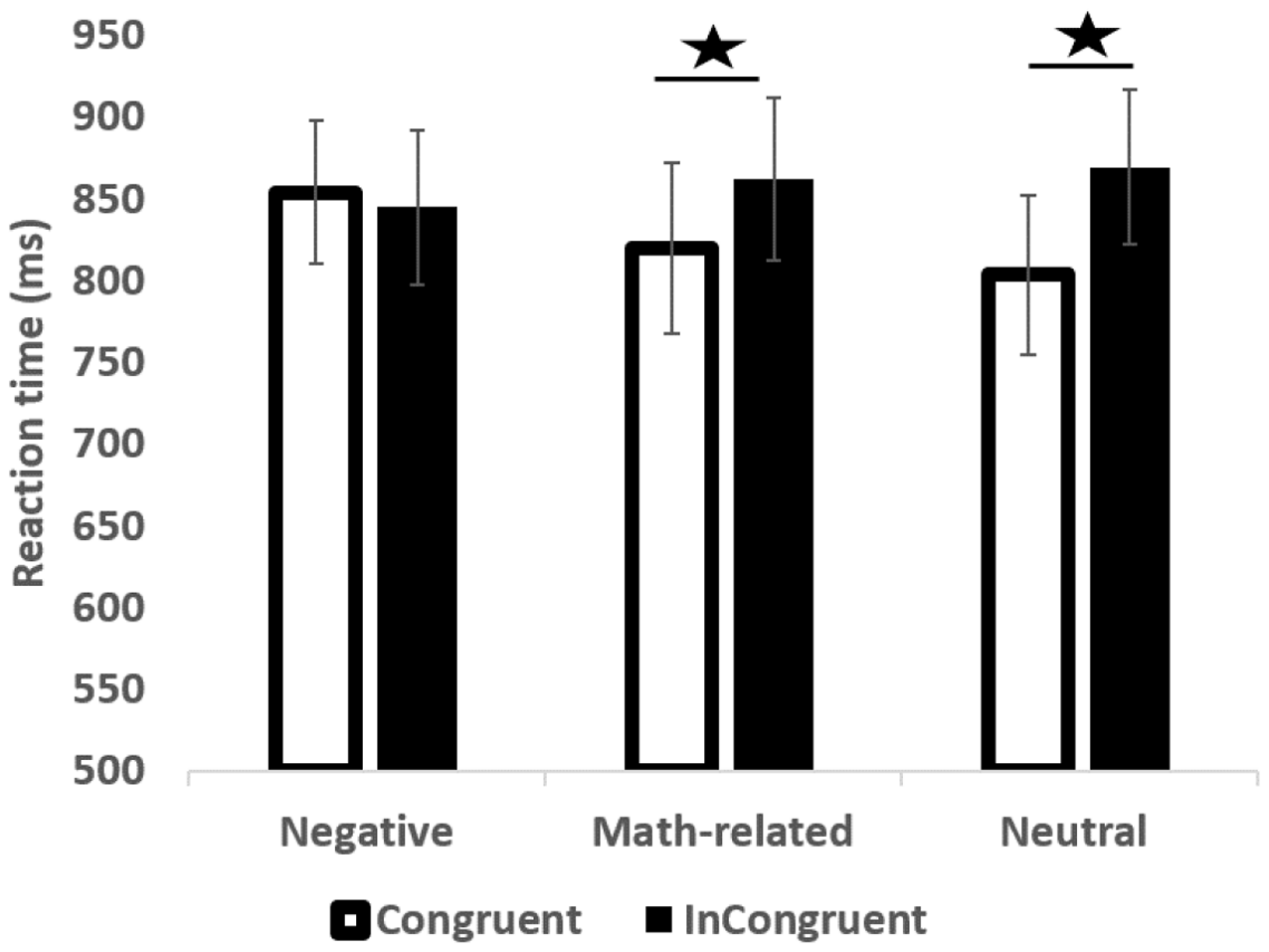
Reaction times in the math-related modified flanker 0task as a function of distractor valence (neutral, negative and math related), and congruency (congruent, incongruent). * < 0.05, error bars represent standard errors.

**Figure 3:**
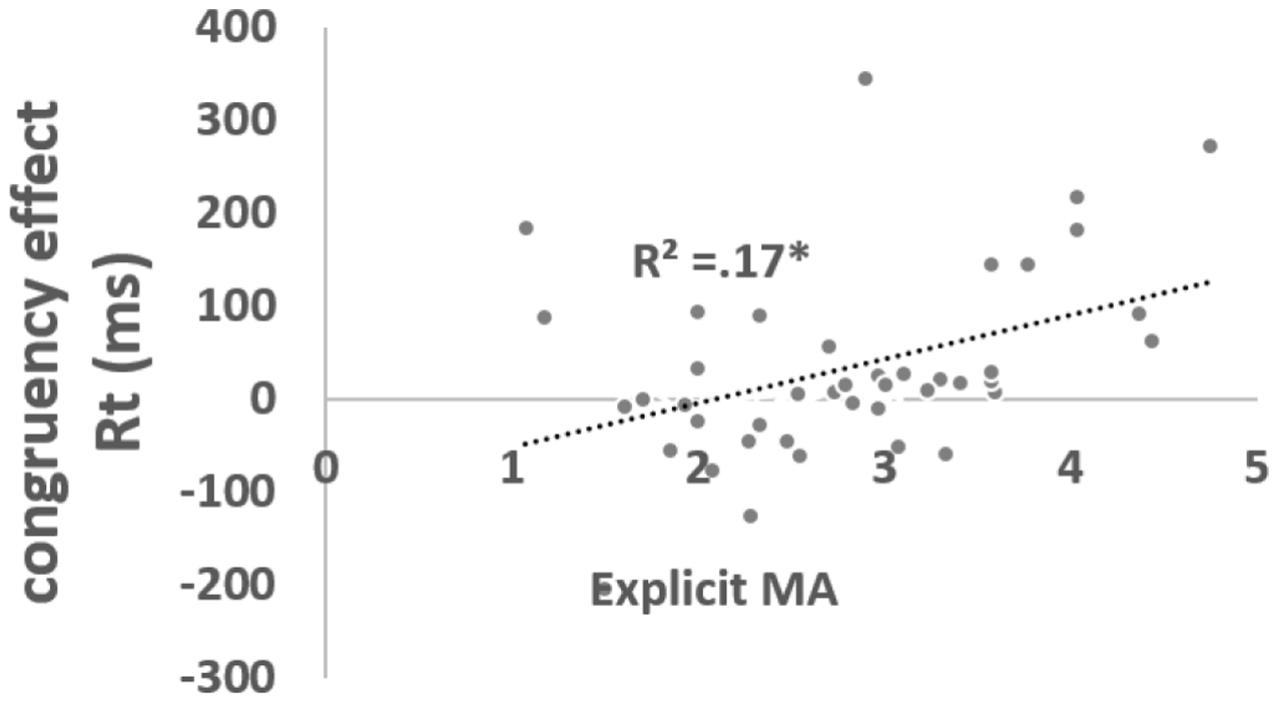
Congruency effect as a function of verbal MA (tested by the MARS).

First, a significant main effect of location congruency was found, *F*(1, 39) = 4.25, *p* < 0.05, partial η^2^ = 0.10, η^2^ = 0.002, VS-MPR 2.60 (see Figure 2). The interaction between congruity and verbal MA (MARS scores) was significant, *F*(1, 39) = 5.48, *p* < 0.05, partial η^2^ = 0.15, η^2^ = 0.003, VS-MPR 4.05 (substantial). The relation between the congruity effect (reaction times for incongruent minus congruent trials) and MARS scores was positive, *r*(40) = 0.41, 95% CI(0.07, 0.71), *p* = 0.007, VS-MPR = 10.61, indicating that individuals with higher verbal MA exhibited a larger congruity effect (see Figure 3).

Second, the interaction between valence and congruity was not significant, *F*(2, 78) = 0.26, *p* = 0.77. However, based on *a priori* hypotheses, we examined congruity in each valence condition. The congruity effect was significant for both neutral pictures (M congruent = 803 ms, SD = 271; M incongruent = 869 ms, SD = 338), *t*(41) = −2.12, *p* = 0.04, 95% CI for mean differences CI 95%(−128, −2.11). Cohen’s *d* = −0.33 CI for Cohen’s d 95%(−0.65, −0.015), VS-MPR = 2.86, and math-related pictures (M congruent = 819 ms, SD = 248; M incongruent = 862 ms, SD = 302), *t*(41) = −2.13, 95% CI for mean differences [−82, −2.1], *p* = 0.04, Cohen’s *d* = −0.33, 95% CI (−0.64, −0.015), VS-MPR = 2.87. However, the congruity effect was not significant for negative pictures (M congruent = 853 ms, SD = 378; M incongruent = 845 ms, SD = 319), *t*(41) = 0.27, *p* = 0.72 (see Figure 2).

**Figure 4:**
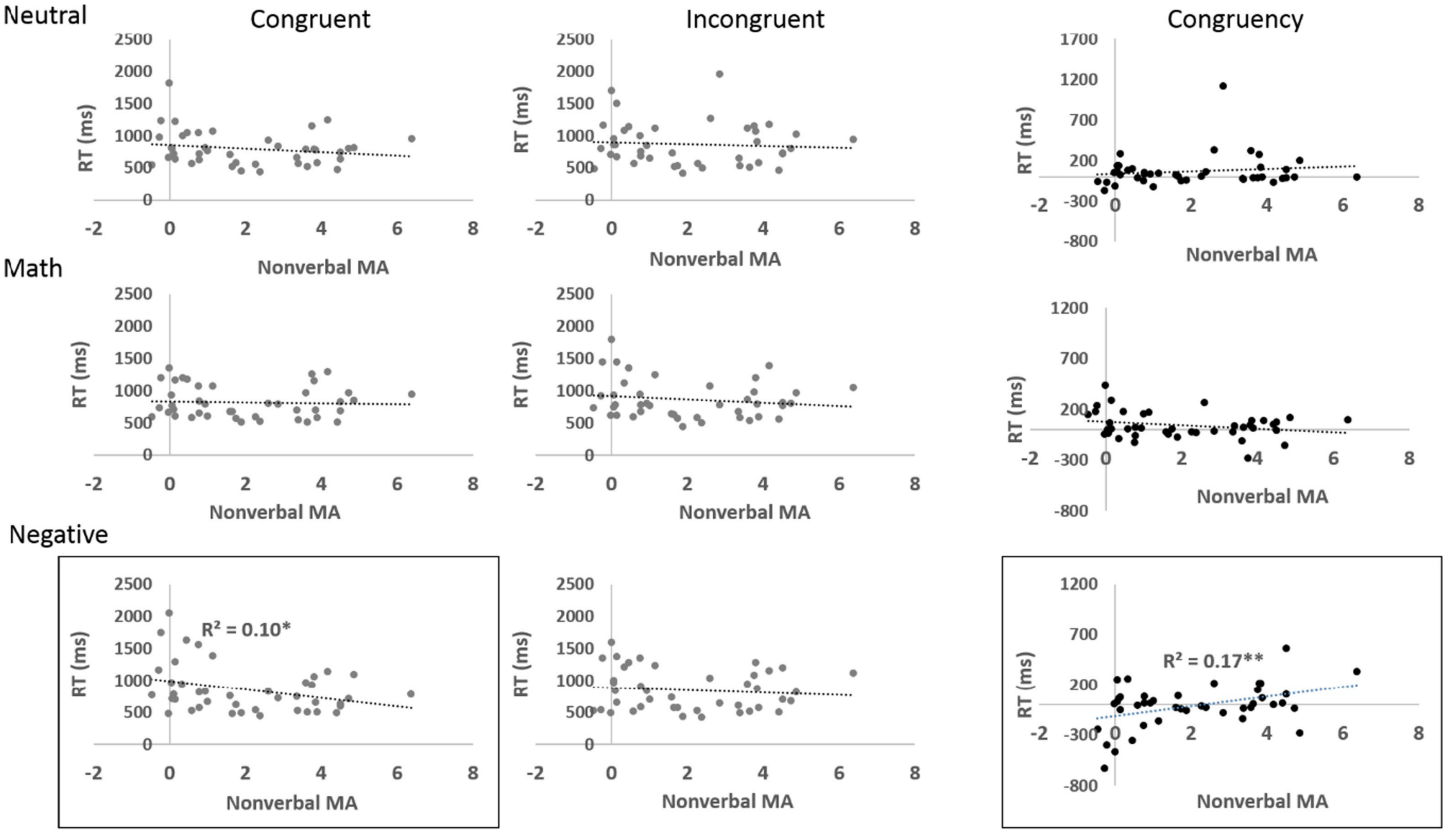
Reaction times in the congruent (left panel), incongruent (middle panel), and congruency conditions (right panel) effect in neutral (top), math (middle), and negative (bottom) valence as a function of nonverbal MA (tested by the negativity of the rating of the math related pictures minus the negativity of the rating of the neutral pictures). Error bars represent standard errors.

**Figure 5:**
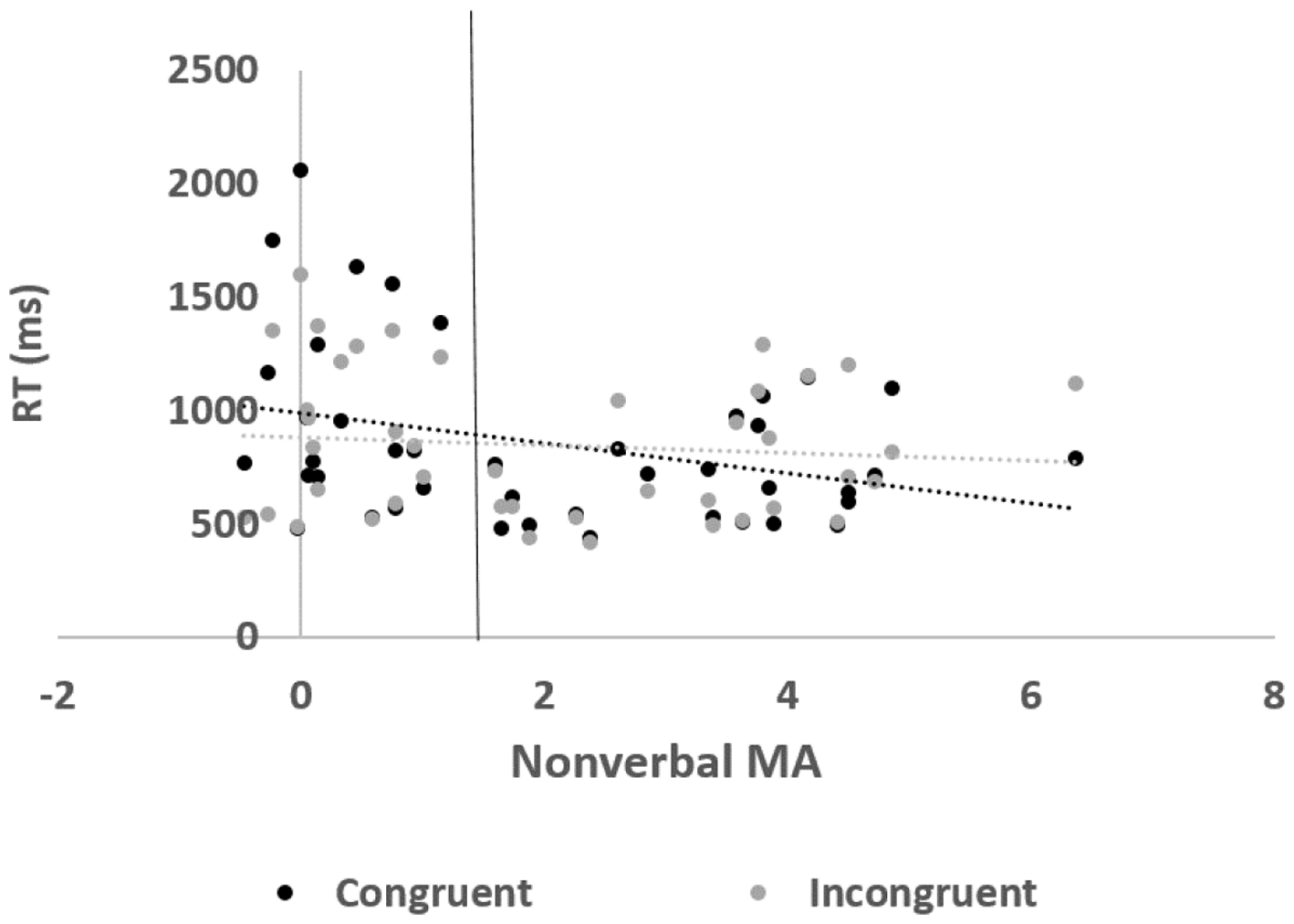
Reaction times in the congruent and incongruent conditions in the negative valence condition as a function of nonverbal MA. The line represents a median split between high and low nonverbal MA.

Third, a significant three-way interaction was observed among *valence*, *congruity*, and nonverbal MA, *F*(2, 78) = 4.84, *p* < 0.05, partial η^2^ = 0.11, η^2^ = 0.007, VS-MPR 7.72 (substantial). Nonverbal MA was negatively correlated with reaction times in the *congruent negative-picture* condition, *r*(40) = −0.32, 95% CI (0.07, 0.65), *p* = 0.039, VS-MPR = 2.896, and positively correlated with the *congruity effect* in the negative-picture condition, *r*(40) = 0.42, 95% CI (0.04, 0.51), *p* = 0.006 VS-MPR = 11.5 (see Figure 4). These findings indicate that participants with low nonverbal MA exhibited a negative congruity effect (i.e., longer reaction times for congruent than incongruent trials), whereas those with high nonverbal MA showed the typical positive congruity effect. To further explore this pattern, participants were divided using a median split on nonverbal MA (high > 1.59; low < 1.59). As expected, the high-nonverbal-MA group showed an average positive congruity effect (*M* = 54 ms, *SD* = 174), whereas the low-nonverbal-MA group exhibited a negative congruity effect (*M* = −86 ms, *SD* = 238). This difference was significant, *t*(40) = −2.21, *p* = 0.03, Cohen’s *d* = −0.684, 95% CI (−1.31, −0.055), Bayesian *t*-test BF10 = 2.012, % error = 0.005 (see Figure 5).

### MRMFT: accuracy analysis

First, the effect of verbal MA (MARS) was significant, *F*(1, 39) = 5.63, *p* < 0.05, partial η^2^ = 0.13. Second, the effect of nonverbal MA (math-related negativity) approached significance, *F*(1, 39) = 3.94, *p* = 0.05, partial η^2^ = 0.09. In both cases, lower anxiety was associated with higher accuracy. None of the remaining main effects or interactions reached significance.

## Discussion

In the present study, we examined the influence of a newly developed set of pictures depicting situations that evoke MA among individuals with HMA, including scenes such as a math classroom with a teacher, a mathematics test, and other related contexts. In a validation study, HMA participants rated math-related pictures as evoking greater anxiety than participants in the LMA group (Ashkenazi et al., 2024). A recent study that used another set of math-related pictures (Linares et al., 2025) found similar results; specifically, the HMA group assessed math-related pictures with lower valence and dominance, and higher arousal, than neutral pictures. Indeed, individuals with HMA emotionally processed the math-related pictures similarly to unpleasant, low-arousing stimuli. Math-related pictures reliably produce heightened anxiety responses in HMA participants, supporting their validity as stimuli for eliciting and assessing nonverbal MA. Unlike previous studies that relied on verbal self-reports, we introduced a nonverbal measure of MA using math-related pictures to better understand the relationship between attentional bias and MA through novel methodologies.

The current study modified the spatial flanker paradigm to examine how MA affects attention to irrelevant math-related, negative, or neutral pictures (Lichtenstein-Vidne et al., 2012; Lichtenstein-Vidne et al., 2017). Given prior research suggesting that individuals with anxiety exhibit biases toward negative stimuli in the emotional Flanker Task (Pizzie and Kraemer, 2017), we sought to address these gaps by incorporating negative valence pictures alongside our newly developed math-related pictures. We aimed to examine both (1) the spatial component of the flanker task and (2) the emotional automatic response to general negative or math-related pictures. Pizzie and Kraemer (2017) discovered a significant location congruity effect for negative stimuli. They found that for participants with verbal HMA, a significant location congruity effect was present in negative stimuli, while for low verbal MA mostly a negative congruity was observed. Consistent with these findings, the current study similarly found that a larger congruity effect across conditions was associated with higher verbal MA in general. Specifically, the location congruity was modulated by valence. A consistent location congruity effect characterized both neutral and math stimuli. However, in negative stimuli, the congruity effect was variable. While some of the participants showed a positive congruity effect, similar to the one in math or neutral stimuli, other participants showed a disengagement bias, evidenced by longer reaction times in the congruent condition compared to the incongruent condition. This suggests difficulty disengaging attention from the negative distractor when it appeared at the attended location. Importantly, this tendency was modulated by nonverbal MA. Participants with low nonverbal MA mostly demonstrated the disengagement bias in negative distractors. High nonverbal MA mostly showed the regular congruity effect (similar to math and neutral pictures), where congruent locations were processed faster than incongruent locations, regardless of distractor valence. This demonstrates that the location congruity effect is much stronger in participants with HMA and is less affected by the valence of irrelevant distractors.

### Spatial abilities in HMA

Analysis of the spatial component of the flanker task revealed a larger location-congruency effect as the level of verbal MA increased when viewing math-related or neutral pictures. This finding demonstrates that attentional or inhibitory control deficits characterize individuals with HMA. We believe that this finding concurs with the spatial theory of MA, which posits a link between MA and spatial weaknesses (Ferguson et al., 2015). Given that the preverbal representation of numbers is based on a spatially represented mental number line (de Hevia, 2016), children with weaker spatial skills may develop less effective preverbal representations of quantities, potentially leading to increased anxiety about mathematics (Ferguson et al., 2015). Supporting this perspective, the current study’s finding of a larger location-congruency effect in the spatial flanker task as the level of MA increases reinforces the idea that spatial weaknesses are present in HMA populations (Ashkenazi et al., 2024).

### The nature of attentional bias in MA

Regarding the emotional component of the spatial flanker task, we aimed to examine whether negative or math-related emotional pictures evoke attentional bias. Previous findings are mixed: some studies indicate that HMA participants show attentional bias toward math-related stimuli (Rubinsten et al., 2015), while others suggest that domain-general attentional mechanisms are disrupted in HMA individuals (Ashkenazi et al., 2024; González-Gómez et al., 2023; Núñez-Peña and Campos-Rodríguez, 2024).

In the present study, we found evidence of a unique effect for negative distractors. First, we found an interaction between congruity and valence. There was no significant congruity effect in the negative condition. Specifically, the congruent condition took longer than the incongruent condition. This pattern is indicative of a disengagement bias: in the congruent condition, participants direct their attention toward the location of the target, and the distracting pictures are processed. In the incongruent condition, the location of the distractors is not in the focus of attention, making it easier to ignore them. Importantly, the interaction between congruity and valence was modulated by nonverbal MA and indicated a disengagement bias toward the negative picture among low nonverbal MA participants. High nonverbal MA participants were better at ignoring the negative picture and showed the regular location congruity effect (similar to math and neutral stimuli). Hence, in line with previous studies (Ashkenazi et al., 2024; González-Gómez et al., 2023; Núñez-Peña and Campos-Rodríguez, 2024), we suggest that high nonverbal MA participants are strongly influenced by spatial attention and do not exhibit domain-specific attentional bias toward math-related or negative stimuli.

### Nonverbal vs. self-report measures of MA

Traditionally, MA has been assessed using explicit self-report questionnaires (Alexander and Martray, 1989; Ashcraft, 2002; Ashcraft and Krause, 2007; Hopko et al., 1998; Suinn and Winston, 2003; Thomas and Dowker, 2000). However, these self-report measures have several limitations. In contrast, nonverbal measures of MA assess participants’ task performance without explicitly inquiring about their MA (Westfall et al., 2021). Studies on general anxiety suggest that nonverbal and explicit anxiety are distinct constructs (Egloff and Schmukle, 2002; Gschwendner et al., 2008). In the current study, participants rated math-related and neutral pictures on three emotional dimensions. Although participants were explicitly asked about anxiety, this measure was less direct than traditional self-report questionnaires. Crucially, the spatial effect of larger congruity was observed using verbal MA self-report measures, whereas attentional bias effects were detected only via the nonverbal MA measurement. This finding further supports the notion that nonverbal and verbal MA are distinct constructs, reinforcing the need for multidimensional assessment approaches.

### Conclusion and limitation

The current study used the Math-Related Modified Flanker Task (MRMFT), a modified version of the spatial flanker task with math-related and negative pictures as distractors, while assessing both nonverbal and verbal MA. Using verbal self-report measurements of MA, we discovered that the location congruity effect was positively related to the level of verbal MA, hinting that MA participants have a general weakness in attentional control that is domain-general and not specific to mathematical stimuli.

Furthermore, the impact of negative valence pictures differed significantly based on nonverbal MA levels. Participants with high nonverbal MA were less affected by negative valence pictures than low nonverbal MA participants. While low nonverbal MA participants showed a disengagement bias toward the negative pictures, high nonverbal MA participants showed the regular location congruity effect regardless of emotional valence. This pattern suggests that a deficit in spatial or executive attention, rather than an attentional bias toward a specific stimulus, is at the heart of MA.

It is important to note, however, that these group differences were found using a small sample size (final *n* = 42) and a median split based on the nonverbal MA measure. Consequently, the absence of attentional bias toward negative stimuli in participants with high nonverbal MA contrasts with some previous studies (Rubinsten et al., 2015; Suárez-Pellicioni et al., 2015). Therefore, alternative explanations should be considered, such as task-specific limitations or limited paradigm sensitivity. Another limitation of the current study is that we assessed general state anxiety, rather than trait general anxiety, and that we tested it following the MRMFT; thus, these scores likely reflect task-induced state anxiety rather than a general disposition. Consequently, this measure does not capture general trait anxiety, which may confound assessments of MA. Future research should explicitly include and control for general trait anxiety as a covariate when investigating MA. A further limitation of this study involves using identical visual stimuli for both the MRMFT and the nonverbal MA assessment. Repeated presentation of the same pictures introduces the risk of habituation or reduced measurement sensitivity during the second exposure. Because we administered the pictures during the MRMFT first, followed by the nonverbal anxiety measure, the primary experimental task was protected from this potential confound. Nonetheless, utilizing distinct stimulus sets for the MRMFT and the nonverbal MA assessment is recommended for future research.

Notably, different results were obtained when using nonverbal versus explicit MA. This finding demonstrates the need to develop alternative tools to assess MA. Furthermore, this study highlights the importance of using both nonverbal and verbal measures to capture the complexities of MA. Gaining a deeper understanding of the nature and underlying mechanisms of MA may help alleviate its symptoms, thereby enabling more students to pursue careers in engineering and other mathematics-related subjects.

The current study had several notable limitations. First, regarding sample size, the sample was small given the use of multiple within-subject factors, continuous covariates, higher-order interactions, and a median split, all of which raise concerns about statistical power. To overcome this limitation, we reported several measures of effect size, confidence intervals, and non-parametric statistics. Moreover, to examine nonverbal MA, we subtracted the neutral ratings from math-related ratings based on the assumption that neutral stimuli provide a stable baseline; however, we did not empirically justify this assumption.

## Data Availability

The raw data supporting the conclusions of this article will be made available by the authors, without undue reservation.

## Footnotes

1
https://www.sona-systems.com/

2 The International Affective Picture System (IAPS) is a set of emotionally evocative color photographs representing a wide variety of events and objects encountered in human experience that have been rated on dimensions of pleasure, arousal, and dominance by men and women. The IAPS pictures are widely used in studies of human emotion worldwide.
