# Supplementary material for: Mathematical anxiety is associated with general rather than specific weakness in attention
Source: Front Psychol. 2026 May 13;17:1763006. doi: 10.3389/fpsyg.2026.1763006 (PMC13212463; doi:10.3389/fpsyg.2026.1763006)
Supplement: Supplementary file 1 [file Table_1.docx]

Table S1. Mean score (across 3 scales) of the math related and neural picture by participants

| **Participant number** | **Math rating** | **Naturel rating** | **Math related negativity** | |
| --- | --- | --- | --- | --- |
| 1 | 5.09 | 1.34 | 3.75 | |
| 2 | 5.42 | 2.07 | 3.35 | |
| 3 | 5.61 | 1.21 | 4.4 | |
| 4 | 1.84 | 1.09 | 0.75 | |
| 5 | 3.46 | 1.08 | 2.38 | |
| 6 | 1.18 | 1.65 | -0.47 | |
| 7 | 5.83 | 1.33 | 4.5 | |
| 8 | 3.21 | 2.20 | 1.01 | |
| 9 | 2.15 | 2.05 | 0.1 | |
| 10 | 6.49 | 1.77 | 4.72 | |
| 11 | 1.63 | 1.05 | 0.58 | |
| 12 | 5.18 | 5.12 | 0.06 | |
| 13 | 2.24 | 1.78 | 0.46 | |
| 14 | 5.34 | 5.32 | 0.02 | |
| 15 | 1.38 | 1.24 | 0.14 | |
| 16 | 7.46 | 3.62 | 3.84 | |
| 17 | 2.96 | 1.37 | 1.59 | |
| 18 | 8.77 | 6.18 | 2.59 | |
| 19 | 1.44 | 1.11 | 0.33 | |
| 20 | 7.36 | 3.55 | 3.81 | |
| 21 | 1.24 | 1.27 | -0.03 | |
| 22 | 6.45 | 2.57 | 3.88 | |
| 23 | 1.23 | 1.18 | 0.05 | |
| 24 | 4.63 | 1.01 | 3.62 | |
| 25 | 1.42 | 1.54 | -0.12 | |
| 26 | 2.70 | 1.92 | 0.78 | |
| 27 | 2.07 | 1.30 | 0.77 | |
| 28 | 1.20 | 1.43 | -0.23 | |
| 29 | 3.31 | 1.43 | 1.88 | |
| 30 | 7.19 | 2.33 | 4.86 | |
| 31 | 6.20 | 2.04 | 4.16 | |
| 32 | 1.88 | 1.11 | 0.77 | |
| 33 | 3.60 | 2.45 | 1.15 | |
| 34 | 2.05 | 2.06 | -0.01 | |
| 35 | 3.31 | 1.04 | 2.27 | |
| 36 | 3.14 | 3.42 | -0.28 | |
| 37 | 2.79 | 1.06 | 1.73 | |
| 38 | 4.76 | 2.77 | 1.99 | |
| 39 | 1.14 | 1.09 | 0.05 | |
| 40 | 1.18 | 1.04 | 0.14 | |
| 41 | 2.93 | 1.28 | 1.65 | |
| 42 | 8.88 | 2.51 | 6.37 | |
| 43 | 6.92 | 3.54 | 3.38 | |
| 44 | 4.36 | 1.51 | | 2.85 |
| 45 | 8.25 | 3.75 | | 4.5 |
| 46 | 1.26 | 1.08 | | 0.18 |
| 47 | 5.09 | 1.34 | | 3.75 |
| 48 | 5.42 | 2.07 | | 3.35 |
| **Mean (S.D.)** | 3.93 (2.31) | 2.03 (1.20) | | 1.90 (1.82) |
